# The subtle balance of trust: how employees’ expected and perceived trust influence impression management

**DOI:** 10.3389/fpsyg.2025.1526860

**Published:** 2025-04-11

**Authors:** MinMin Zhang, Xiaodong Ma

**Affiliations:** ^1^The Army Infantry College of PLA, Shijiazhuang, Hebei, China; ^2^Business School, Central University of Finance and Economics, Beijing, China

**Keywords:** expected trust, perceived trust, pro-social impression management, pro-self impression management, cubic response surface analysis

## Abstract

**Introduction:**

This study examines how employees expected and perceived trust influence impression management strategies, focusing on their interplay in shaping pro-social and self-oriented behaviors. Trust is pivotal in organizations, yet research has overlooked the impact of trust mismatches on impression management. Addressing this gap, we analyze the effects of trust congruence and explore behavioral variations under different trust combinations.

**Methods:**

Using a quantitative approach, we analyze survey data from employees across various enterprises. To test the hypotheses, we first conducted polynomial regression analysis, followed by response surface analysis. The primary polynomial regression aims to test the hypotheses of ascendant ridge, consistency, and asymmetry, further revealing the complex relationships between the variables.

**Results:**

Trust congruence fosters pro-social impression management and curtails self-oriented behaviors. Higher trust levels correlate positively with pro-social behaviors. Notably, trust incongruence has asymmetric effects: employees with high expected but low perceived trust resort to self-oriented strategies, while those with low expected but high perceived trust exhibit stronger pro-social tendencies.

**Discussion:**

These findings highlight the need to promote trust congruence in management. Addressing trust misalignment through tailored strategies, such as enhancing communication and support, can foster constructive behaviors. Future research should explore trust dynamics and moderating organizational factors like leadership and culture. This study advances understanding of workplace trust and offers practical insights for management.

## 1 Introduction

In recent years, as work models have increasingly shifted towards flexibility and remote arrangements, traditional mechanisms of leader control have gradually weakened, leading to a significant rise in the need for leaders to trust their employees (Wohlers and Hertel, 2017). Simultaneously, the growing complexity of work makes it difficult for leaders to possess sufficient expertise in all areas, further heightening their reliance on employees’ professional capabilities (Humphrey et al., 2007). As a result, trust has become particularly crucial in leader-subordinate relationships (Dirks and de Jong, 2022). Trust is commonly defined as a willingness to be vulnerable in the presence of others (Mayer et al., 1995; Rousseau et al., 1998), and it is a core element of effective leadership. This is especially true in modern work environments, where leaders must depend on the judgment and skills of their employees.

Moreover, trust is not merely an attitude that leaders hold toward their employees; it also exerts influence through employees’ perceptions. When employees perceive trust from their leaders, the “feeling of being trusted” emerges (Lau et al., 2007). This represents a vital social and emotional resource, signaling to employees that they are valued and positively regarded within the organization (Lau et al., 2014). In turn, this feeling can stimulate their work motivation and sense of responsibility. Research has shown that perceived leader trust enhances job satisfaction (Goris et al., 2003; Colquitt et al., 2007; Ugwu et al., 2016; Legood et al., 2021), task performance (Lau et al., 2014), and proactive work behaviors (Lester and Brower, 2003), while also contributing to the development of high-performing teams (Lau et al., 2007; Salamon and Robinson, 2008).

Although existing research on the effects of perceived leader trust is relatively well-developed, in practice, leader trust in employees often manifests as specific behaviors, such as relying on their judgment and abilities or depending on their assistance in critical matters (Spreitzer and Mishra, 1999; Brower et al., 2000). Therefore, while being trusted can bring benefits, it also places high demands on employees’ time and energy (Baer et al., 2015, 2021). These conflicting conclusions regarding the effects of perceived leader trust not only pose challenges for academic research but also create dilemmas for managers in practical application. Consequently, further exploration of the effectiveness and underlying mechanisms of perceived leader trust is of significant importance for both theoretical advancement and managerial practice.

According to person-environment fit theory, employees’ perceptions and responses are shaped not only by their individual characteristics but also closely linked to their organizational environment (Edwards, 1996). When employees’ needs align with perceived organizational factors, such as leadership style or organizational support, they tend to exhibit more positive attitudes and behaviors. Conversely, a misalignment between their needs and perceptions may lead to negative emotions, stress, or dissatisfaction. Future research should therefore further investigate the alignment between employees’ needs and their perceived leader trust and its effects on employee behavior and attitudes. To address this research gap, Baer et al. (2021), drawing on fairness theory, examined the alignment between employees’ expected trust (trust expectations) and the actual trust they receive (trust perceived) and its effects on employees’ fairness perceptions and job performance (Baer et al., 2021). These studies have revealed that when employees’ trust expectations align with the trust they actually receive, they perceive greater fairness and demonstrate improved job performance. However, this fairness-based perspective primarily focuses on the alignment between trust supply and demand, yet is limited in explaining the relationship between employees’ role positioning within the organization, role expectations, and behavior. Within the framework of role theory, employees not only have expectations of their own but also play specific roles shaped by the organization’s and leaders’ expectations. Role theory suggests that employees’ behaviors are influenced by the roles they assume and leaders’ expectations of these roles (Katz and Kahn, 1978). Thus, employees may not only respond to discrepancies in trust based on fairness perceptions but also be influenced by their role perception, leaders’ definition of their roles, and their understanding of these role expectations. In other words, the alignment between trust expectations and trust perceived may affect not only fairness perceptions but also employees’ understanding of their roles within the organization, thereby influencing their behaviors and performance.

Secondly, this study primarily focuses on employees’ resources and characteristics, without adequately considering relational factors between leaders and employees. While prior research has examined the top-down effects of leaders’ trust behaviors on employees (Song et al., 2023), it has overlooked the bottom-up influence that employees exert on leaders. Trust, as a critical component of leader-employee relationships, is not built instantaneously but is gradually established and strengthened through mutual interaction (Nienaber et al., 2023; Yang and Tsai, 2023). Through everyday communication, collaboration, and interdependence, trust accumulates over time. In this process, leaders convey trust to employees by assigning responsibilities, empowering them with autonomous decision-making, and providing support and feedback. In response, employees reciprocate by fulfilling their roles, demonstrating professional competence, and maintaining reliability, thereby reinforcing mutual trust. Furthermore, research has shown that employees can influence leaders’ trust behaviors through impression management strategies, a mechanism that warrants further exploration in future studies.

In organizational environments, employees face pressures to gain leaders’ trust and recognition, often leading them to adopt impression management strategies (Weber et al., 2004; Campagna et al., 2020). Research indicates that employees typically use two primary strategies under these pressures: pro-self impression management and pro-social impression management (Sosik et al., 2002; Peck and Hogue, 2018). Pro-self impression management focuses on emphasizing personal achievements and abilities to strengthen leaders’ trust in their individual competencies, while pro-social impression management prioritizes team collaboration and collective interests to gain support and acceptance from both leaders and colleagues. These strategies reflect different needs and motivations that employees experience in the workplace.

Our study, grounded in role theory, explores how the relationship between employees’ expected leader trust and perceived leader trust influences their choice of impression management strategy. When there is alignment between expected and perceived leader trust, this consistency fosters an increase in pro-social impression management while reducing the need for pro-self impression management. In such cases, employees experience psychological security, which encourages them to show support and cooperation with the team. However, where expected and perceived leader trust align in a linear manner, as expected trust increases, employees may simultaneously increase both pro-self and pro-social impression management, indicating a balance between personal and team-oriented image-building efforts. Conversely, when there is a mismatch between expected and perceived trust, strategy choices vary. If expected trust exceeds perceived trust, employees may tend toward pro-self impression management; if expected trust is lower than perceived trust, employees may shift toward pro-social impression management as a means to repair the trust relationship.

In summary, this study makes three key contributions. First, it advances research on employee impression management in the trust domain by distinguishing between pro-self and pro-social impression management strategies. Existing literature often treats impression management as a singular construct, focusing on how employees gain leader trust by emphasizing positive behaviors or masking negative ones. However, this oversimplified view overlooks the multidimensional nature of impression management, where distinct strategies arise from different motivations and yield varied outcomes. By introducing pro-self and pro-social strategies, this study addresses a critical theoretical gap, offering a nuanced perspective on how employees influence leader trust through diverse impression management approaches. Second, adopting a role theory perspective, this study sheds light on the complex relationship between employees’ role expectations and leader trust—an aspect overlooked by fairness-based research. Fairness theory primarily conceptualizes trust through perceptions of input–output fairness, assuming stable role expectations in leader-employee interactions. However, trust dynamics involve evolving and subjective role perceptions, influencing employees’ behavioral responses. This study highlights that when there is a misalignment between expected and perceived leader trust, employees strategically employ impression management (pro-self or pro-social) to signal role expectations and realign trust perceptions. This perspective extends trust research by uncovering the intricate signaling processes within leader-employee trust interactions, which fairness theory fails to capture. Finally, this study enriches the dynamic perspective of trust research by examining leader trust alignment and its impact on employees’ impression management strategies. Trust is often treated as a static variable, with limited attention to its evolving nature and behavioral consequences. By analyzing the consistency, linearity, and misalignment between expected and perceived leader trust, this study demonstrates that trust is not fixed but continuously adjusted based on employees’ perceptions and leaders’ behaviors. Employees actively respond to trust fluctuations by selecting impression management strategies that align with trust dynamics, shaping subsequent work behaviors and performance. This bidirectional and interactive perspective offers a more comprehensive understanding of organizational trust, emphasizing its fluidity and strategic implications.

## 2 Theory and hypothesis development

### 2.1 Trust perceived and trust expected

Trust is defined as an individual’s positive expectation regarding others’ intentions, capabilities, and integrity, prompting reliance on others and a willingness to accept potential risks in uncertain situations (Mayer et al., 1995; Rousseau et al., 1998). Currently, trust is understood from two perspectives. One perspective frames trust as a psychological state characterized by positive expectations about others’ motives and behaviors. This viewpoint is grounded in subjective assessments of ability, benevolence, and integrity (Mayer et al., 1995), emphasizing the emotional and cognitive factors that shape internal feelings of trust (McAllister, 1995). The other perspective considers trust as a behavior or tendency, focusing on actions within specific contexts. In this view, trust is manifested through behavior in particular situations or formed by observing others (Lau et al., 2007), highlighting its role in social interactions (Brower et al., 2000, 2009).

Despite the varying perspectives on trust, the focus shifts depending on research objectives. Specifically, studies of trust typically emphasize the psychological state of the trustor, while research on being trusted concentrates on the behaviors that convey trustworthiness to the trustee (Baer et al., 2021). Building on this distinction, we define perceived trust as the degree to which an employee perceives their supervisor as engaging in behaviors that reflect trust in the employee. For instance, when employees are frequently solicited for input on significant projects, entrusted to represent their supervisor in meetings, or relied upon for their expertise and abilities, they interpret these actions as indicators of their supervisor’s trust (Gillespie, 2011). Conversely, employees who are seldom asked to participate in critical tasks may perceive a lower level of trust from their supervisor (Baer et al., 2021).

When a project manager expects their supervisor to rely on their professional judgment and skills for important decisions, rather than depending solely on reports from other departments, and anticipates being granted autonomy throughout the project’s implementation, this reflects a request for trust (Skinner et al., 2014). Trust expected refers to the extent to which employees expect their supervisors to exhibit trust behaviors. This includes the expectation that supervisors will rely on their judgment, skills, and abilities for important matters.

In organizations, leaders and employees interact through their respective roles, each holding role expectations that shape their behavior and communication. Employees develop expectations of their leaders based on their need for trust, which may include providing clear instructions, supporting teamwork, and resolving conflicts. When leaders meet these expectations, trust is reinforced; when they fail to do so, trust diminishes, adversely affecting work attitudes and performance. Individual differences, such as background and personality, also influence how both parties form and interpret these expectations. Misalignment can lead to role conflict and erode trust. When employees feel misunderstood, they may resort to impression management strategies to convey their needs and expectations.

### 2.2 Trust perceived, trust expected, and impression management

Impression management refers to the behaviors employed by employees, known as actors, to shape the perceptions of others—typically their supervisors and colleagues, but also potentially including employees and clients—in the workplace (Bolino et al., 2016). This process involves creating a expected image or maintaining and protecting an existing one through various tactics, including self-promotion, ingratiation, exemplification, intimidation, and supplication (Bolino and Turnley, 1999; Bolino et al., 2008).

The generation of expectations is a fundamental psychological function that underpins individual behavioral motivation (Leary and Kowalski, 1990; Merkl-Davies and Brennan, 2011; Johnson et al., 2016). When individuals believe they can achieve expected outcomes through successful performance, these expectations stimulate their motivation to act, driving them toward their ideal goals. Impression management, as a goal-directed behavior, can be categorized into two orientations: pro-self orientation, which maximizes personal benefits, and pro-social orientation, which seeks to maximize benefits for both oneself and others (Peck and Hogue, 2018). While goal orientation is often viewed as a stable preference (Van Lange, 1999), research indicates that situational factors can influence individuals to engage in either pro-self or pro-social behaviors (Smeesters et al., 2003). Therefore, our focus is on understanding the situational contexts that give rise to employees’ goal orientation preferences, rather than the inherent stability of these orientations.

Importantly, the pro-self or pro-social orientation of impression management strategies is determined not by the strategies themselves, but by the employees’ underlying goals. This means that employees may utilize the same impression management tactics to achieve different outcomes depending on their motivations (Peck and Hogue, 2018). For instance, if an employee employs impression management strategies to gain personal benefits or enhance their self-image, this reflects a pro-self orientation. Conversely, if the intention is to promote the team or assist others, this aligns with a pro-social orientation.

#### 2.2.1 Trust perceived, trust expected, and pro-self impression management

The pro-self impression management strategy focuses on projecting an ideal self-image for the individual (Van Lange, 1999). Such behaviors can emerge as inherent personality traits or be triggered by specific social contexts (Van Lange et al., 1997). When a particular situation activates an individual’s identity recognition, they may engage in self-centered actions. For instance, during job interviews, candidates often employ self-promotion and ingratiation to shape perceptions of their competence and likability (Peck and Levashina, 2017). Thus, pro-self impression management strategies typically arise in distinct contexts.

According to role theory, individuals within organizations encounter role expectations from others, particularly from leaders, and adjust their behavior in response to these expectations. In a trust-related context, the behaviors exhibited by leaders can be seen as expressions of their role expectations for their employees. When the trust behaviors received by employees do not align with their expectations, they may resort to instrumental behaviors, including pro-self impression management, to cope with this misalignment.

We can distinguish between scenarios where there is a lack of perceived trusting behaviors and those where the trust individuals perceive aligns with their expected level of trust. In cases where the trust perceived by employees is lower than their expected level of trust, a situation of deficient trust emerges. Here, unmet role expectations can cause employees to feel neglected by their leaders in terms of attention and support. Role theory suggests that when individuals’ role expectations are misunderstood or unfulfilled, they may engage in instrumental behaviors to communicate their needs (Katz and Kahn, 1978). In situations of deficient trust, employees might employ pro-self impression management behaviors to influence their leaders’ perceptions and demonstrate their worthiness for greater trust. For example, they may emphasize their accomplishments, display loyalty, or exhibit heightened enthusiasm to reinforce their leaders’ sense of trust in them.

Next, we consider the implications of perceived excess trust compared to the fit between expected and perceived trust. While trust is generally regarded as a positive attribute, excessive trust can generate additional stress or discomfort (Baer et al., 2021). Employees may feel that their leaders have set unreasonably high expectations or may be uncertain about their ability to uphold such levels of trust. Role theory posits that employees will react to these heightened role expectations, potentially engaging in pro-self impression management behaviors to manage or meet their leaders’ expectations and avoid disappointing them. For instance, they might strive to enhance their image by appearing more competent or loyal, ensuring that their leaders’ high trust levels remain intact (Jin et al., 2022).

In contrast, when the level of trust expected aligns with the perceived level of trust, there is a strong fit between role expectations, fostering a more stable trust relationship. This congruence in trust suggests that leaders have a clear understanding of their employees’ needs, facilitating smooth role interactions and preventing the behavioral pressures that can arise from either deficient or excessive trust. In situations of trust matching, individuals are more likely to express their authentic selves rather than engage in pro-self impression management aimed at adjusting or influencing others’ perceptions. In such cases, the need for impression management diminishes, as the trust level is appropriate, and the expectations and role interactions of both parties are consistent. Accordingly, we propose the following hypothesis:

Hypothesis 1: Incongruence between the level of trust expected and the level of trust perceived will be positively associated with pro-self impression management. Specifically, individuals will engage in more pro-self impression management when they experience either a deficiency or excess of trust compared to what they expect, reflecting a congruence effect.

The role theory posits that individuals’ expectations and interpretations of roles are influenced by individual differences. When employees have a high expect for trust in their leader and perceive that the leader provides sufficient trust (a match between high perceived trust and high expected trust), they may feel that the leader has high expectations of them. This high level of trust may motivate employees to engage in pro-self impression management behaviors to further solidify their positive image in the eyes of the leader, as they want to ensure they perform exceptionally well in a high-trust relationship. In contrast, when both perceived trust and expected trust are low, interactions between the leader and employees may be rather lukewarm, and employees’ motivation may be weak, leading them to invest less effort in managing their image in front of the leader. Therefore, pro-self impression management behaviors are stronger in states of high perceived and high expected trust.

Individual differences play a crucial role here. Different employees may adjust their behaviors based on their characteristics (such as self-confidence, intrinsic motivation, and sensitivity to feedback) (Cheng et al., 2023). For instance, some employees may expect recognition from their leader more than others; in a high-trust environment, they may engage more actively in pro-self impression management, while others might behave more passively in a trusting context (Ispas et al., 2014). Thus, individual differences can influence how they respond to trust and the level of engagement in impression management. As a result, we propose the following hypothesis:

Hypothesis 2: A deficiency in the trust perceived compared to the expected trust will result in increased pro-self impression management, with stronger effects observed as the level of deficiency escalates, reflecting a linear, rising-ridge effect.

Role theory posits that discrepancies between expected and actual role interactions lead to pronounced behavioral responses (Katz and Kahn, 1978). In situations characterized by low perceived leader trust and high expected leader trust, the widening trust gap heightens employees’ need for behavioral adjustment, compelling them to engage in active impression management. Conversely, in contexts where perceived leader trust is high but expected trust is low, individuals perceive less urgency regarding trust, resulting in a smaller trust gap and reduced motivation for impression management (Legood et al., 2021).

When employees recognize a strong need for trust, they view it as a crucial resource that influences their expectations, growth opportunities, and performance (Dirks and Ferrin, 2002). A lack of sufficient trust from their leader (low perceived trust) may drive these employees to take proactive steps to alter their leader’s perceptions. According to role theory, when individuals feel that their role expectations are misunderstood or unmet, they may resort to instrumental behaviors to communicate their needs and influence others’ expectations. Thus, in circumstances of low perceived trust and high expected trust, employees are more inclined to engage in impression management to reshape their leader’s trust and close the trust gap (Bolino et al., 2016).

In contrast, in scenarios where perceived leader trust is high but expected leader trust is low, employees may feel they already possess ample trust from their leader. Due to their diminished need for trust, they perceive no significant trust gap, resulting in a lack of motivation to engage in additional pro-self impression management (Gillespie and Dietz, 2009; Dirks et al., 2022). Instead, they may choose to maintain their current behavior without actively attempting to alter or reinforce their leader’s perception of them, as their low need for trust does not necessitate further effort to gain more (Norman et al., 2010). Based on the preceding analysis, we propose the following hypothesis:

Hypothesis 3: Employees experiencing a deficiency in leader trust compared to their expected trust will engage in more pro-self impression management than those experiencing an excess of trust, reflecting an asymmetric effect.

#### 2.2.2 Trust perceived, trust wanted and pro-social impression management

Pro-social impression management strategies are not only aimed at helping others, but they also bring benefits to the individual (Monyei et al., 2022). For instance, in work settings, pro-social behaviors can manifest in teamwork, such as when a customer service team works together towards a common goal (Grant and Mayer, 2009). These behaviors also surface in personal interactions, such as offering assistance or emotional support to colleagues. By fostering cooperation and a positive environment, pro-social actions benefit both the organization and its members, creating a harmonious atmosphere that supports overall well-being and productivity (Bolino, 2003). Moreover, research indicates that cultural norms emphasizing social responsibility, cooperation, and supportive behaviors can motivate employees to engage in pro-social impression management. For example, Krupka and Weber (2009) found that cultural norms encourage pro-social behaviors by prompting individuals to reflect on their actions and observe those of others, reinforcing a cycle of supportive interactions (Krupka and Weber, 2009).

Role theory suggests that individuals within organizations engage with one another based on shared role expectations (Biddle, 1986; Anglin et al., 2022). When employees have specific expectations regarding leader trust, and leaders demonstrate a corresponding level of trust through their actions, the mutual exchange of trust becomes a crucial element of role interaction (Cropanzano and Mitchell, 2005). When individuals’ expectations align with their perceptions of their roles, they are more likely to exhibit behaviors that reflect those expectations (De Jong et al., 2016). This alignment fosters a more cohesive working environment and enhances overall organizational effectiveness.

When employees’ perceived leader trust aligns with their expected trust, the match between role expectations and reality is complete. This alignment indicates that employees’ trust needs are met, and their role expectations are accurately understood and addressed by their leaders. According to role theory, when expectations and perceptions are congruent, individuals are more inclined to sustain or enhance this positive interactive relationship, leading to increased job satisfaction and organizational commitment (Dirks and Ferrin, 2002; De Jong et al., 2016). Furthermore, the reciprocal nature of trust fosters a supportive work environment, which is essential for team performance and overall organizational effectiveness (Gillespie and Mann, 2004; Cropanzano and Mitchell, 2005).

In scenarios where trust is aligned, employees are more inclined to engage in pro-social impression management behaviors. This alignment fosters a sense of belonging and responsibility, motivating employees to contribute positively (Colquitt et al., 2007). By practicing pro-social impression management, employees further strengthen their trust relationship with their leaders, which not only helps maintain existing trust but also enhances their image in the eyes of the leader (Kim et al., 2018).

Conversely, when trust is lacking, employees may diminish their engagement in pro-social impression management. Without the support of trust, they might feel that their efforts will go unrewarded, leading to decreased commitment to the organization or leader. In essence, unmet trust needs can result in reduced positive behaviors, as employees lack the motivation to enhance their image in others’ eyes (Lapierre and Hackett, 2007).

Additionally, in situations of excessive trust, employees may also scale back their pro-social impression management behaviors. Feeling over-trusted, they might perceive no need to further enhance their leader’s perception of them through additional positive actions. Consequently, they may consider the existing level of trust sufficient, rendering further efforts to improve their image unnecessary (Schaubroeck et al., 2011). Given this understanding of trust dynamics, we propose the following hypothesis:

Hypothesis 4: The alignment between perceived leader trust and expected leader trust will positively influence pro-social impression management. Specifically, individuals will engage in more pro-social impression management when their perceived trust level aligns with their expected trust level, demonstrating a congruence effect.

Role theory suggests that employees’ behavior aligns with their role expectations within an organization, often shaped by mutual trust dynamics between leaders and employees. When high trust alignment is present, employees experience role clarity, which motivates them to engage in pro-social behaviors such as impression management (Colbert and Witt, 2009). In such contexts, employees feel that their trust expectations are met, fostering a collaborative and constructive environment (Sluss and Ashforth, 2008).

However, in scenarios where trust alignment is low, employees may perceive a lack of recognition or feel undervalued, which can dampen their motivation to engage in behaviors that exceed basic role requirements, such as pro-social impression management (Podsakoff et al., 2006). This lack of trust alignment can lead to reduced engagement and potentially to role ambiguity, diminishing employees expect to maintain or enhance their image proactively. Given this understanding of trust alignment through the lens of role theory, we can anticipate distinct employee behaviors based on varying levels of trust congruence. When trust between leaders and employees aligns at high levels, it not only meets role expectations but also fosters a mutual reinforcement of positive behaviors. Conversely, when trust alignment is low, employees may feel less motivated to engage in additional positive actions, as they perceive limited value in doing so. Building on these insights, we propose the following hypothesis:

Hypothesis 5: Higher perceived leader trust and expected leader trust will positively influence pro-social impression management to a greater degree than lower levels of trust alignment, exhibiting a linear, rising-ridge effect.

When the trust that employees expect from their leader does not align with their actual perceived trust, it can lead to varied behavioral responses. We will explore two specific scenarios: one where perceived trust is high but trust expectations are low, and another where perceived trust is low but trust expectations are high.

In the first scenario, employees anticipate low trust from their leader but perceive a higher-than-expected level of trust. This positive mismatch can create a pleasant surprise for employees, reflecting a favorable outcome overall. According to role theory, because their expectations are either met or exceeded, employees are unlikely to suffer significant negative consequences. They may continue to engage in pro-social impression management, driven by the security and support derived from the unexpected higher trust. While some pro-social behaviors might diminish—due to a perceived reduced need to enhance their image—the overall negative impact remains minimal (Grant and Sumanth, 2009; Carattini and Roesti, 2023).

Conversely, in the second scenario, employees expect a high level of trust but perceive a lower-than-expected level. This negative mismatch can lead to frustration and dissatisfaction, as unmet expectations diminish their sense of value and recognition within the organization. As a result, employees may withdraw from pro-social impression management behaviors, feeling that further efforts would not alter their leader’s perception. The resulting disappointment and frustration can weaken their motivation, further straining the relationship with their leader (Wang et al., 2021). Thus, we propose the following hypothesis:

Hypothesis 6: Compared to scenarios of high perceived leader trust with low expected leader trust, the combination of low perceived leader trust and high expected leader trust will exert a stronger negative effect on pro-social impression management, reflecting an asymmetric effect.

## 3 Materials and methods

### 3.1 Sample and procedure

We tested our hypotheses based on a sample of 330 employee-supervisor pairs from three companies in the service, finance, and technology sectors in northern China. To ensure the validity of the sample, we considered the following factors when selecting companies. First, we reviewed the companies’ official websites and communicated with management to ensure that their organizational structures and cultures are highly dependent on the trust relationship between leaders and employees, with trust significantly influencing employee behavior. Second, companies whose primary business does not involve a high degree of teamwork or interpersonal interaction were excluded from consideration, as the impact of trust on employees’ pro-social and pro-self behaviors may not be sufficiently evident in such environments. Third, the selected companies must have clear management hierarchies and communication channels to ensure that employees’ pro-social and pro-self impression management behaviors can be clearly perceived by their leaders, thereby enhancing the accuracy of our measurements of the interaction between trust and behavior.

Our data collection process was conducted in several structured steps. First, we engaged with HR managers to communicate the study’s objectives—specifically, to examine how perceived leader trust and the consistency of expected leader trust influence pro-social and pro-self impression management. We provided clear guidelines on the operational procedures and sought their assistance in identifying eligible line managers for participation. Next, HR managers reached out to line managers, who were instructed to randomly select one to four eligible subordinates from a provided name list. This random selection process helped ensure a representative sample and minimize selection bias. Additionally, line managers completed a supervisor questionnaire to assess the performance of the selected employees, allowing us to incorporate their subjective evaluations into our analysis. Once the list of eligible employees was finalized, all selected participants were gathered in a designated meeting room with the support of HR managers. They were thoroughly briefed on the study’s purpose and its contribution to understanding the dynamics of leader trust and employee behavior. To encourage candid responses, we assured participants of the confidentiality of their answers, emphasizing that their input would be used solely for academic research.

Employees provided data on their demographic variables, perceived leader trust, and expected leader trust, while their supervisors rated their pro-self and pro-social impression management. To facilitate the matching of subordinate responses with their supervisors’ evaluations, each questionnaire was assigned a unique identification number. To ensure confidentiality and response reliability, participants were given envelopes to seal their completed questionnaires. This design aimed to enhance their sense of privacy protection, encouraging more candid expressions of their views and feelings. The entire data collection process was carefully monitored to ensure voluntary and anonymous participation, thereby enhancing the authenticity and credibility of the research data.

In summary, we sent out 400 questionnaires to 80 frontline managers and received 330 valid responses from 73 frontline managers and their 330 subordinate employees, resulting in a response rate of 82.5% for employees and 91.25% for frontline managers. Among the 330 employees, 56.4% were male and 43.6% were female. In terms of age, 70.3% were 30 years old or younger, while 29.7% were between 30 and 40 years old. Regarding education, 60% held an associate degree, while 40% held a bachelor’s degree or higher.

### 3.2 Measures

We began by selecting internationally recognized scales to measure all variables in this study. Next, we applied a translation-back-translation method to ensure the accurate translation of all English-based measurement tools. Finally, we utilized a consistent response scale across all measurement tools, specifically a five-point Likert scale ranging from 1 (strongly disagree) to 5 (strongly agree).

#### 3.2.1 Pro-self impression management

Self-promotion can serve as an effective tool for measuring pro-self impression management, as it directly involves behaviors aimed at influencing others’ perceptions by highlighting one’s strengths and achievements. Pro-self impression management refers to a range of strategies individuals use to control or guide others’ views of them, and self-promotion is a common and specific method to achieve this. Therefore, self-promotion is a key behavior in pro-self impression management, particularly in situations where emphasizing personal abilities and value is crucial, as it effectively reflects an individual’s pro-self motivation. Ultimately, we chose self-promotion to measure pro-self impression management. Specifically, pro-self impression management was measured by self-promotion using a four-item scale originally developed by Bolino and Turnley (1999). Cronbach’s alpha was 0.918.

#### 3.2.2 Pro-social impression management

It is reasonable to use exemplification as an indicator of pro-social impression management because it demonstrates an individual’s pro-social orientation through a series of concrete actions. For instance, behaviors such as selfless assistance, integrity, and high-performance standards not only shape a person’s image within the organization but also contribute to fostering a cooperative team culture. As such, exemplification serves as an effective measure of pro-social impression management. Through these actions, individuals not only exhibit their contributions to others and the organization, thereby crafting a pro-social image, but also enhance teamwork and promote the maximization of collective benefits. This dual effect places exemplification at the core of pro-social impression management, making it a key indicator of pro-social tendencies. We selected exemplification to measure pro-social impression management, specifically, pro-social impression management was measured by exemplification using a five-item scale originally developed by Gardner and Cleavenger (1998). Cronbach’s alpha was 0.869.

#### 3.2.3 Perceived trust and expected trust

Perceived trust was measured using the five-item scale developed by Baer et al. (2021), with a Cronbach’s alpha of 0.900. Expected trust was also measured using the five-item scale developed by Baer et al., with a Cronbach’s alpha of 0.910.

### 3.3 Research methods

First, we first conducted a common method bias (CMB) test. Next, we conducted confirmatory factor analysis (CFA) to assess the validity of the key variables. To test the hypotheses, we then performed polynomial regression analysis, followed by response surface analysis (Edwards, 1994; Humberg et al., 2019). The primary polynomial regression was designed to capture the rising ridge, congruence, and asymmetry (RRCA) hypotheses (Humberg et al., 2022). A second-order polynomial can only test simple congruence hypotheses, as its function is always parabolic and symmetric. Evaluating more complex asymmetrical hypotheses requires more sophisticated polynomial regressions. The linear rising ridge enables us to analyze the main effects of predictor variables, in contrast to the “strict” model, which only accounts for congruence effects without linear effects. The RRCA model is a special case of the full third-order polynomial model, with specific parameter constraints that reflect the hypothesized relationships.

## 4 Research result

### 4.1 Common method bias test

To address the potential influence of common method bias (CMB) on the results, we first employed Harman’s single-factor test to examine whether common method bias exists in the data. The results indicated that four factors with eigenvalues greater than one were extracted, accounting for 72.646% of the total variance. The variance explained by the first factor was 27.842%, which did not exceed 50% of the total variance. This suggests that the data does not suffer from a single factor explaining the majority of the variance, implying that common method bias is not severe (Podsakoff and Organ, 1986). To further ensure that common method bias would not pose a problem, we followed the unmeasured latent method construct (ULMC) technique recommended by Williams and McGonagle (2016). According to this test, we added a ULMC factor to the baseline four-factor model and compared the fit indices of the two models (Williams and McGonagle, 2016). The results showed that the baseline model (χ^2^/df = 1.216, CFI = 0.992, TLI = 0.990, SRMR = 0.033, RMSEA = 0.026) did not significantly improve after adding the ULMC factor to form the new model (χ^2^/df = 1.567, CFI = 0.979, TLI = 0.975, SRMR = 0.055, RMSEA = 0.041). Therefore, there is no common method bias (CMB) issue in this study.

### 4.2 Discriminant validity test of variables

In this study, confirmatory factor analysis was conducted using Mplus 8.3 to test the four constructs: expected trust, perceived trust, pro-self impression management, and pro-social impression management (see Table 1). The results indicate that the data fit indices of the four-factor model are superior to those of alternative models, demonstrating that the four-factor model has good discriminant validity.

**Table 1:** Confirmatory-factor analysis.

Model	χ2	df	RMSEA	CFI	TLI	SRMR
Five factors	227.271	145	0.041	0.979	0.975	0.055
Four factors	177.530	146	0.026	0.992	0.990	0.033
Three factors (1)	902.987	149	0.124	0.804	0.775	0.135
Three factors (2)	1010.835	149	0.132	0.776	0.742	0.128
Two factors	1723.323	151	0.178	0.590	0.536	0.179
One factor	2973.427	152	0.237	0.265	0.173	0.260

Five-factor model: Pro-self impression management, pro-social impression management, perceived trust, expected trust, ULMC. Four-factor model: Pro-self impression management, pro-social impression management, perceived trust, expected trust. Three-factor model (1): Combined pro-self impression management and pro-social impression management, perceived trust, expected trust. Three-factor model (2): Combined perceived trust and expected trust, pro-self impression management, pro-social impression management. Two-factor model: Combined pro-self impression management and pro-social impression management, combined perceived trust and expected trust. One-factor model: All variables combined.

### 4.3 Descriptive statistics and correlation analysis

We conducted descriptive statistical analysis on the mean and standard error of each variable, as well as correlations between variables. As shown in Table 2, the correlations between variables are consistent with our theoretical expectations, providing preliminary support for the hypotheses of this study.

**Table 2:** Means, standard deviations, and correlations between variables.

Variables	*M*	SD	1	2	3	4
1. Expected trust	3.270	1.016	1			
2. Perceived trust	3.381	0.984	0.386**	1		
3. Pro-self impression management	3.508	1.138	0.153**	−0.151**	1	
4. Pro-social impression management	2.560	0.880	0.032	0.249**	−0.142**	1

*N* = 330. *p < 0.05; **p < 0.01.

### 4.4 Hypotheses tests

We conducted three response surface analyses in the R environment (version 4.4.1) using the RSA package (version 0.10.6). Specifically, in the first step, we tested model constraints on the full third-order model to examine whether there is a significant difference between the RRCA model and the full model. In the second step, we tested whether the correlation coefficients were statistically significant. Finally, in the third step, we conducted an inspection of the range of realistic predictor combinations.

#### 4.4.1 Pro-self impression management

The results are shown in the Table 3 below. The broad asymmetric congruence model did not fit the data significantly worse than the full model (χ2 = 10.653, *p* = 0.100), indicating that the data supports the broad asymmetric congruence model. Therefore, the broad asymmetric congruence model can be used for subsequent data analyses and hypothesis testing. Cohen (1988) introduced the effect size measure f^2^ for applications in multiple regression, hierarchical regression, and analysis of variance. Following the analytical approach of Humberg and Nestler, multiple regression was employed to test the proposed hypotheses. The results show that the f^2^ value for the RRCA model is 0.23. According to Cohen (1988), this effect size indicates that the model explains a considerable proportion of the variance in the dependent variable, highlighting the practical significance of the examined relationships within the regression framework (Cohen, 1988).

**Table 3:** Cubic response surface analysis results for the pro-self impression management.

Model	*b*_0_	*b*_1_	*b*_2_	*b*_3_	*b*_4_	*b*_5_	*b*_6_	*b*_7_	*b*_8_	*b*_9_	*u*_1_	∆χ^2^	*R* ^2^
RRCA model	3.184	0.033	0.033	0.305	−0.610	0.305	0.104	−0.312	0.312	−0.104	0.065	10.653	0.187
*p*-value	0.000	0.426	0.426	0.000	0.000	0.000	0.000	0.000	0.000	0.000	0.426	0.100	
Full model	3.351	0.286	−0.318	0.215	−0.624	0.226	0.023	−0.113	0.162	0.002	−0.033		0.213
*p*-value	0.000	0.070	0.048	0.000	0.000	0.001	0.698	0.221	0.076	0.982	0.872		

RRCA model = broad asymmetric congruence model. Full model = full third-order polynomial model.

**Figure 1:**
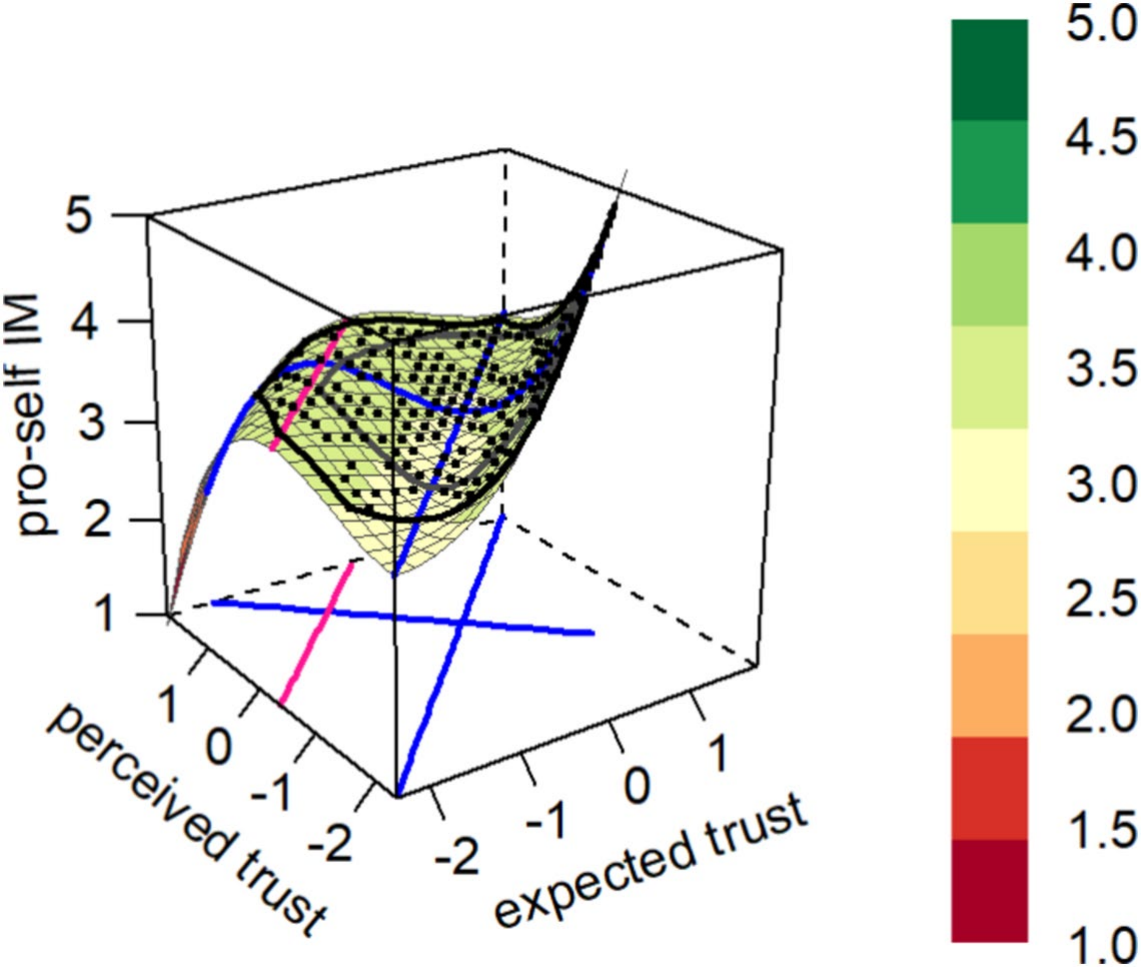
Graph of the estimated rising ridge asymmetric congruence model for the pro-self impression management.

The consistency between employees’ expected leader trust and perceived leader trust has a negative effect on pro-self impression management (*b*_3_ = 0.305, *p* < 0.001). The hypothesized positive effect of high perceived leader trust and high expected leader trust on pro-self impression management was not supported when compared to low perceived leader trust and low expected leader trust (*u*_1_ = 0.065, *p* > 0.05). However, compared to high perceived leader trust and low expected leader trust, low perceived leader trust combined with high expected leader trust had a stronger positive effect on pro-self impression management (*b*_6_ = 0.104, *p* < 0.001). As shown in the figure, the positive coefficient means that, beginning at the LOC, the surface rises faster in the direction of incongruence where “expected > perceived” than in the direction of “expected < perceived.” Finally, there were no predictor combinations positioned beyond the second extremum line E2 (as shown by the pink line in the Figure 1). Thus, Hypothesis 1 and Hypothesis 3 are supported (Figure 1).

The coefficient estimates b_0_ to b_9_ refer to the full third-order polynomial model:


$$\begin{array}{l} z=b_{0}+b_{1}x+b_{2}y+b_{3}x^{2}+b_{4}xy+b_{5}y^{2}\\ \;+b_{6}x^{3}+b_{7}x^{2}y+b_{8}xy^{2}+b_{9}y^{3} \end{array}$$

where x = expected trust, y = perceived trust, z = pro-self impression management. u_1_ = coefficient of the linear level effect in the RRCA model, computed as $u_{1}=b_{1}+b_{2}$ .

∆χ2 = difference between the values of the two models.

#### 4.4.2 Pro-social impression management

**Figure 2:**
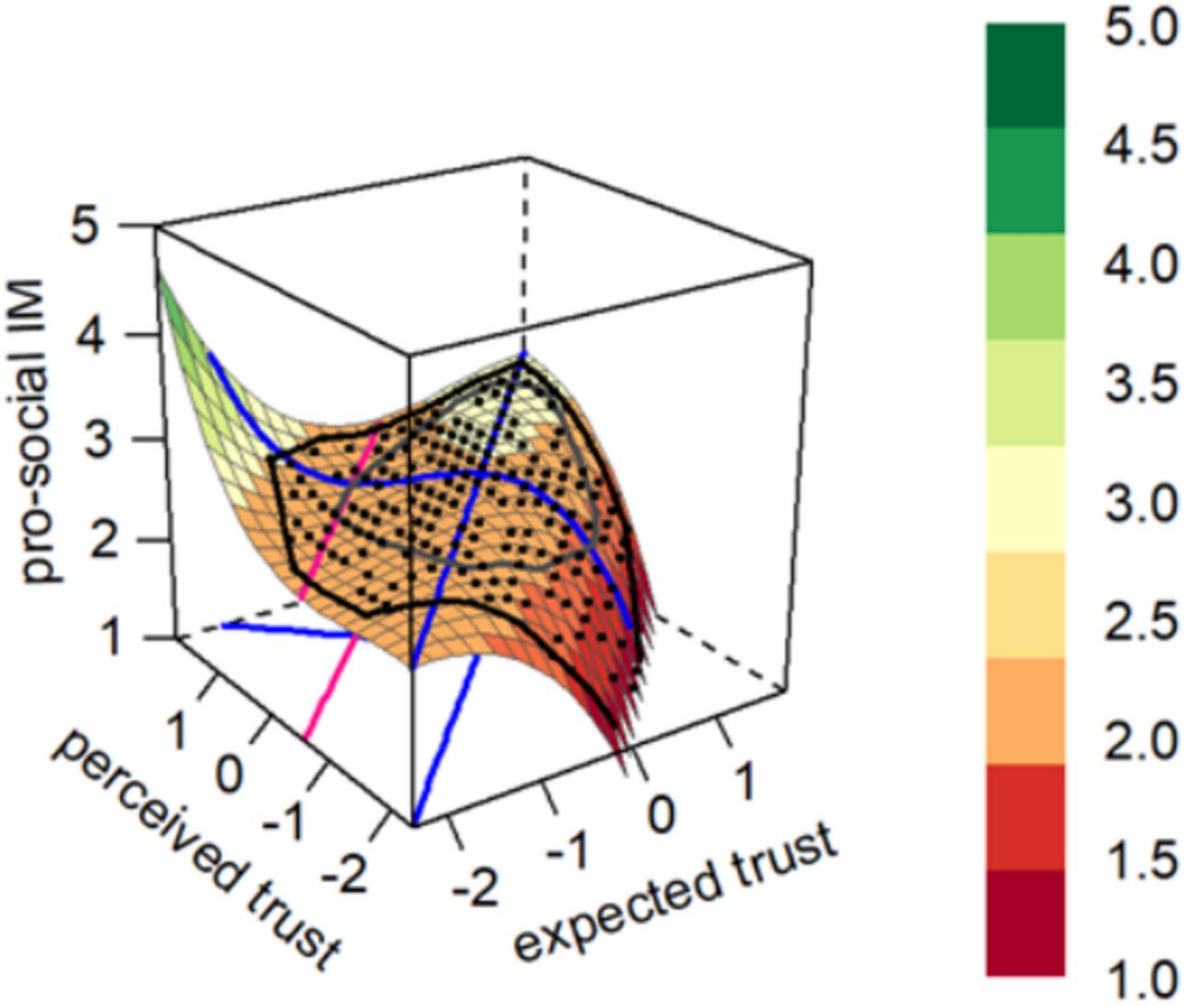
Graph of the estimated rising ridge asymmetric congruence model for the pro-social impression management.

The broad asymmetric congruence model did not fit the data significantly worse than the full model (χ2 = 8.754, *p* = 0.188), suggesting that the data supports the use of the broad asymmetric congruence model (see Table 4). The results show that the *f*^2^ value for the RRCA model is 0.189. The consistency between employees’ expected leader trust and perceived leader trust has a positive effect on pro-social impression management (*b*_3_ = −0.188, *p* < 0.001). The hypothesized positive effect of high perceived leader trust combined with high expected leader trust on pro-social impression management was confirmed when compared to low perceived leader trust and low expected leader trust (*u*_1_ = 0.141, *p* < 0.01). Furthermore, when compared to the combination of low perceived leader trust and high expected leader trust, high perceived leader trust combined with low expected leader trust had a stronger positive effect on pro-social impression management (*b*_6_ = −0.071, *p* < 0.001). As illustrated in the figure, the negative coefficient indicates that starting from the line of congruence (LOC), the surface declines more sharply in the direction of incongruence where “expected > perceived” than where “expected < perceived.” Lastly, although 0.3% of predictor combinations were positioned beyond the second extremum line E2 (shown by the pink line in Figure 2), they still fall within an acceptable range. Hypothesis 4, Hypothesis 5, and Hypothesis 6 are thus supported (Figure 2).

**Table 4:** Cubic response surface analysis results for the pro-social impression management.

Model	*b*_0_	*b*_1_	*b*_2_	*b*_3_	*b*_4_	*b*_5_	*b*_6_	*b*_7_	*b*_8_	*b*_9_	*u*_1_	∆χ^2^	*R* ^2^
RRCA model	2.755	0.070	0.070	−0.188	0.376	−0.188	−0.071	0.214	−0.214	0.071	0.141	8.754	0.159
*p*-value	0.000	0.009	0.009	0.000	0.000	0.000	0.000	0.000	0.000	0.000	0.009	0.188	
Full model	2.756	0.301	0.083	−0.255	0.400	−0.121	−0.179	0.234	−0.260	0.081	0.384		0.179
*p*-value	0.000	0.020	0.481	0.000	0.000	0.017	0.000	0.001	0.000	0.091	0.011		

RRCA model = broad asymmetric congruence model. Full model = full third-order polynomial model.

The coefficient estimates b_0_ to b_9_ refer to the full third-order polynomial model:


$$\begin{array}{l} z=b_{0}+b_{1}x+b_{2}y+b_{3}x^{2}+b_{4}xy+b_{5}y^{2}\\ \;+b_{6}x^{3}+b_{7}x^{2}y+b_{8}xy^{2}+b_{9}y^{3} \end{array}$$

where x = expected trust, y = perceived trust, z = pro-social impression management. u_1_ = coefficient of the linear level effect in the RRCA model, computed as $u_{1}=b_{1}+b_{2}$ .

∆χ2 = difference between the values of the two models.

### 4.5 Empirical findings

#### 4.5.1 Trust incongruence and pro-self impression management

The findings indicate that when employees’ expected trust and perceived trust are misaligned, pro-self impression management significantly increases. Compared to trust congruence, employees in incongruent trust situations are more likely to engage in strategic self-presentation to adapt to their environment, whereas trust congruence reduces such behavior. Further analysis reveals that high trust congruence (high perceived–high expected trust) does not significantly enhance pro-self impression management compared to low trust levels. This suggests that merely being in a high-trust environment does not inherently drive employees to engage in self-presentation, as high trust alone is not a decisive factor in shaping impression management behaviors. Additionally, trust deficits (expected trust exceeding perceived trust) are more likely to trigger pro-self impression management than trust surpluses (perceived trust exceeding expected trust). When employees perceive a gap between their expected and actual received trust, they are more inclined to adopt strategic behaviors to influence leadership perceptions, highlighting the negative impact of trust incongruence.

Overall, trust incongruence, particularly trust deficits, significantly increases employees’ engagement in pro-self impression management. However, high trust congruence does not necessarily amplify such behavior. These findings underscore the need for leaders to not only acknowledge employees’ perceived trust but also align it with their trust expectations. Reducing trust gaps can mitigate employees’ reliance on strategic self-presentation, fostering more authentic and stable leader-employee relationships.

#### 4.5.2 Trust incongruence and pro-social impression management

The results suggest that trust congruence enhances employees’ engagement in pro-social impression management. When expected and perceived trust align, employees are more likely to demonstrate positive behaviors proactively rather than strategically adjust their image to conform to leadership expectations. The sense of security and stability derived from trust congruence enables employees to focus on organizational goals. Further analysis shows that high trust congruence (high perceived–high expected trust) is more effective in stimulating pro-social impression management than low trust congruence. Employees who both expect to be trusted and perceive strong trust from their leaders are more inclined to reciprocate through positive behaviors, reinforcing a culture of mutual trust. However, trust deficits exert a stronger negative impact on pro-social impression management than trust surpluses. When employees’ trust expectations are not met, their willingness to engage in pro-social behaviors declines, weakening trust interactions within the organization.

These findings highlight the critical role of trust congruence in fostering employees’ proactive behaviors. Organizations should not only cultivate trust but also ensure alignment between employees’ trust expectations and their actual perceptions. Minimizing trust gaps can prevent employees from disengaging due to trust incongruence, ultimately promoting a stable and sustainable trust culture within the organization.

## 5 Discussion

### 5.1 Theoretical implication

This study broadly contributes to the trust literature in the following aspects. First, the traditional view holds that when employees feel more trusted by their supervisors, they tend to perform better (Salamon and Robinson, 2008; Brower et al., 2009; Lau et al., 2014). However, recent studies suggest that such trust can also lead to pressure, which some employees may struggle to handle (Skinner et al., 2014; Baer et al., 2015, 2021). Therefore, the key lies in whether the “expected trust” and the “perceived trust” are aligned, rather than the absolute level of trust itself (Baer et al., 2021). Only when these are in alignment will employees’ perceptions of fairness be positively influenced, which in turn enhances performance. Trust arises from the interpersonal interactions between leaders and employees, meaning that employees are not passively receiving the level of trust from their leaders or merely experiencing psychological reactions (Wong, 2019). Instead, they may engage in upward influence behaviors, such as impression management strategies. By examining employees’ reactions to whether leaders understand their trust expectations, this study aims to contribute to the trust literature from a bottom-up perspective. This expands the traditional interpersonal view of trust and challenges the implicit assumption that employees remain passive in the trust process, merely reacting to trust-related decisions. This represents a significant shift in trust theory. Research on employees’ upward influence behaviors in response to trust could offer new insights into the reciprocal nature of power dynamics and potentially open up broader areas for trust research.

Second, this study expands the intersection of trust and impression management. While existing research predominantly focuses on how employees’ impression management behaviors influence leader trust (Weber et al., 2004; Campagna et al., 2020), our study takes a different approach by investigating how different combinations of expected leader trust and perceived leader trust, in turn, affect employees’ impression management strategies. This offers a new framework for trust research, highlighting that trust is not a unidirectional behavior but rather a complex, bidirectional interaction process. Moreover, prior studies often overlook the differences in types of impression management (Gardner and Martinko, 1988; Bolino, 2003; Bolino et al., 2008; Al-Shatti and Ohana, 2021; Parker and Schmitz, 2022). By categorizing these types, this research offers deeper insights into the motives behind employees’ use of these strategies and how leaders respond to them, enriching the theoretical lens of trust research. Exploring the potential drivers of both pro-self and pro-social impression management, particularly under different combinations of trust expected and perceived, uncovers the psychological mechanisms underlying employees’ choices of these strategies. These findings not only advance theoretical understanding but also provide practical implications for management by helping managers better identify and respond to employees’ impression management behaviors.

Third, this study introduces role theory as a theoretical foundation to explain how employees’ perceived and expected trust levels influence their impression management strategy decisions. Role theory helps to understand how employees respond to role expectations from supervisors or colleagues within the organization (Biddle, 1986). In an effort to adjust others’ expectations and impressions, employees may exhibit specific behaviors, such as instrumental behaviors. The application of this theory reveals how employees respond behaviorally to different role expectations. Furthermore, role theory emphasizes the importance of individual differences in the formation and interpretation of role expectations (Eagly and Karau, 2002; Anglin et al., 2022). Building on this foundation, psychological contract theory (PCT) and social exchange theory (SET) provide complementary insights into employees’ behavioral mechanisms under trust incongruence. Psychological contract theory suggests that discrepancies between expected and perceived trust influence employees’ sense of psychological contract fulfillment or breach, which in turn shapes their behavioral responses. When perceived trust exceeds expectations, employees may engage in prosocial behaviors as a form of reciprocation. Conversely, when perceived trust falls short, they may adopt self-enhancement strategies to compensate for the trust deficit and restore their standing. Social exchange theory extends this perspective by explaining how employees navigate leader-employee trust relationships under conditions of trust asymmetry. Employees who receive greater trust than expected may strengthen prosocial behaviors to maintain relational balance, while those experiencing a trust shortfall may employ self-presentation tactics to reinforce their credibility and reliability in the eyes of their leaders. In summary, this study centers on role theory while incorporating psychological contract theory and social exchange theory to reveal how trust congruence influences employees’ impression management strategies, offering a more comprehensive understanding of trust dynamics.

### 5.2 Practical implications

This study explores how trust misalignment influences employees’ impression management behaviors and provides concrete managerial recommendations based on the findings. These recommendations aim to help organizations mitigate the negative effects of trust misalignment and foster a healthy organizational trust culture.

First, enhancing the alignment of leader trust is essential for improving team collaboration and the organizational climate. Managers should engage in regular communication and feedback processes to accurately assess employees’ expected trust levels and their actual perceived trust. Timely adjustments to management strategies can help reduce the adverse effects of trust misalignment on employee behavior.

Second, implementing trust management training can enhance managers’ awareness of trust dynamics. Through systematic training programs, managers can more precisely identify employees’ trust expectations and adjust their leadership approaches accordingly, thereby minimizing strategic impression management behaviors resulting from trust misalignment.

Furthermore, establishing a structured feedback mechanism can effectively reduce the negative consequences of trust-related information asymmetry. Organizations should develop comprehensive feedback systems that include regular communication, bidirectional feedback, and transparent performance evaluations. This approach enables employees to gain a clear understanding of their trust standing within the organization, thereby reducing the likelihood of engaging in strategic self-presentation behaviors.

In addition, clarifying role expectations can help mitigate trust misalignment caused by role ambiguity. By explicitly defining employees’ responsibilities, leadership expectations, and organizational trust standards, managers can alleviate employees’ uncertainty regarding trust perceptions, allowing them to focus on work contributions rather than relying on impression management strategies.

Finally, establishing trust-based communication mechanisms is crucial for fostering stable leader-employee trust relationships. Managers should proactively engage in trust dialogues with employees, such as through regular one-on-one meetings or team discussions. These interactions allow employees to express their trust expectations while enabling managers to communicate their trust in employees explicitly. Such mechanisms help reduce discrepancies in trust perceptions, minimize unnecessary strategic self-presentation behaviors, and promote more stable and sustainable leader-employee trust relationships.

By implementing these managerial practices, organizations can effectively mitigate the negative consequences of trust misalignment, encourage employees to exhibit more pro-social behaviors, and ultimately enhance team performance and organizational effectiveness.

### 5.3 Limitations and future research

Despite the valuable insights offered by this study, several limitations should be considered when interpreting the findings. This study explores how trust mismatch influences employees’ impression management based on role theory. However, role theory primarily explains behavioral adjustments in response to external expectations, without fully capturing the psychological and long-term effects of trust mismatch. Employees experiencing trust mismatch may perceive psychological contract breaches, affecting their career development and organizational commitment. Future research could integrate psychological contract theory and social exchange theory to explore its impact on long-term behaviors such as commitment and turnover. Additionally, distinguishing cognitive and affective trust mismatches may clarify their effects on impression management strategies.

Moreover, cultural context may limit the generalizability of findings. In China’s high power distance and collectivist culture, employees tend to adopt submissive strategies, aligning with leadership expectations. In contrast, employees in low power distance, individualistic cultures (e.g., Western countries) may employ assertive self-presentation or direct communication. Future research should conduct cross-cultural comparisons and examine whether cultural values, such as power distance and individualism–collectivism, moderate trust mismatch effects.

Furthermore, this study employs cross-sectional data. However, trust evolves dynamically, and employees may adjust their impression management strategies over time. Initially, they may conform to leadership expectations, but prolonged mismatch could lead to disengagement or turnover. Future research should adopt longitudinal designs or experience sampling methods (ESM) to track these behavioral changes and examine contextual factors like leadership transitions or performance evaluations.

In addition, self-reported surveys introduce potential social desirability bias, as employees may overestimate their adaptability or underreport negative impression management tactics (e.g., responsibility avoidance, error concealment). Future research should use multi-source data, including leader and peer evaluations and behavioral observations, to improve validity. Experimental studies could simulate trust mismatch scenarios to directly observe employee responses, enhancing external validity.

Additionally, the study focuses on service, finance, and technology sectors, where trust is crucial. However, in structured industries like manufacturing, healthcare, or government institutions, rigid regulations may limit impression management behaviors, altering the impact of trust mismatch. Future research should explore industry-specific differences and how organizational culture and job stability influence employee responses.

Finally, this study examines trust mismatch’s direct effects but overlooks key moderating factors. Leadership styles (e.g., transformational, ethical leadership) and individual traits (e.g., self-esteem, risk tolerance) may influence employees’ reactions. Future research should incorporate these variables and explore how psychological resilience and emotional regulation shape employees’ ability to adapt to trust mismatch.

## 6 Conclusion

Drawing on role theory, this study examines how trust congruence and incongruence shape employees’ impression management strategies. Based on a two-wave data collection, the findings reveal that employees with trust congruence exhibit a stable increase in pro-social impression management, whereas those experiencing trust incongruence adopt distinct coping strategies. Specifically, when expected trust is lower than perceived trust, employees tend to enhance pro-social behaviors to reciprocate trust. Conversely, when expected trust exceeds perceived trust, employees are more likely to engage in pro-self impression management to compensate for the trust gap. These findings highlight the behavioral implications of trust dynamics, offering new insights into trust management and organizational behavior. Practically, addressing trust misalignment through communication and trust-building initiatives can foster healthier workplace interactions.

## Data Availability

The raw data supporting the conclusions of this article will be made available by the authors, without undue reservation.
